# Development of cannabinoid receptor (CB 2 R) ligands for application in PET studies - where to attach the radiolabel?

**DOI:** 10.1186/1758-2946-6-S1-O9

**Published:** 2014-03-11

**Authors:** Robert Günther, Rareş Moldovan, Corinna Lueg, Winnie Deuther-Conrad, Bernhard Wünsch, Peter Brust

**Affiliations:** 1 Department of Neuroradiopharmaceuticals, Institute of Radiopharmaceutical Cancer Research, Helmholtz-Zentrum Dresden-Rossendorf, Research Site Leipzig, Leipzig, 04368, Germany; 2 Department of Pharmaceutical and Medicinal Chemistry, University of Münster, Münster, 48149, Germany

## 

The cannabinoid receptors type 2 (CB_2_R) are involved in many physiological processes but their expression level in healthy and diseased brain has not been unravelled. With positron emission tomography (PET) it is possible to monitor quantitatively very low amounts of compounds labelled with positron emitting isotopes like ^18^F in living organisms at high spatial resolution. For application in clinical research, such radiotracers have to show high selectivity and affinity to the target protein.

A series of fluorinated *N*-carbazolyl-oxadiazolyl-propionamides [1] was synthesised and the affinity towards the human CB_2_R was measured in receptor binding studies. Here, we combine our CB_2_R receptor model with 3D-QSAR data [2] to support molecular docking studies employing the MOE software (Version 2012.12 Chemical Computing Group Inc. Montreal. http://www.chemcomp.com). The studies revealed that both the primarily investigated compound **2** and the 2-fluoroethyl substituted carbazole derivative **1** (K*_i_* = 3.6 nM) fits well into the binding pocket. Attachment of the fluorine at different positions of the structure does not lead to significantly different poses in accordance with the experimental data. Organ distribution studies on CD1-mice verified our prediction, [3] that [^18^F]1 and [^18^F]2 can cross the blood-brain barrier.

**Figure 1:**
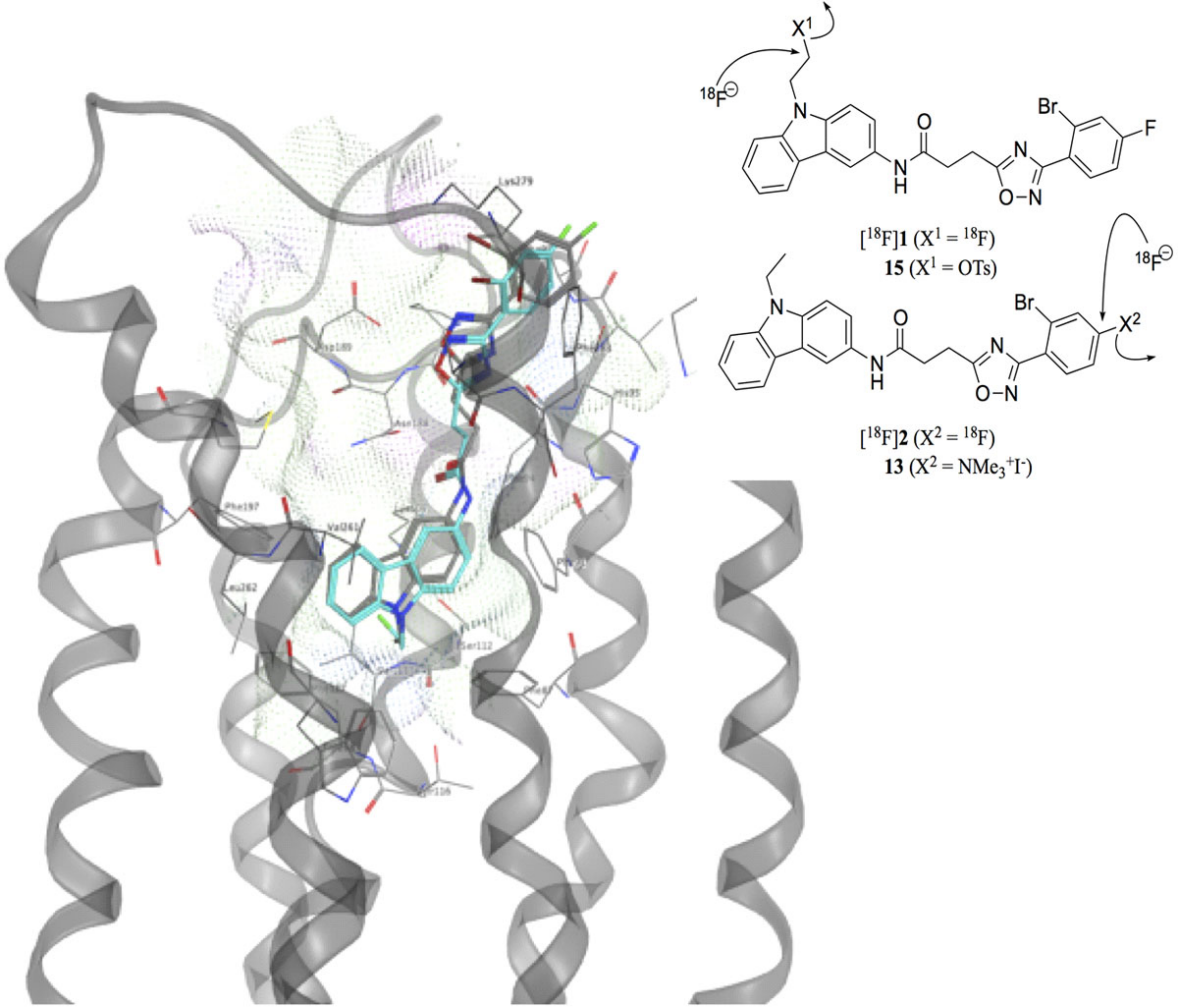
Compounds **1** and **2** fitted into the binding pocket of the CB_2_R receptor model.
